# Exploring alternatives for adolescent anorexia nervosa: adolescent and parent treatment (APT) as a novel intervention prospect

**DOI:** 10.1186/s40337-021-00423-7

**Published:** 2021-06-09

**Authors:** Maria Ganci, Linsey Atkins, Marion E. Roberts

**Affiliations:** 1 APT Therapeutic Solutions, Melbourne, Australia; 2 Department of General Practice & Primary Healthcare, Faculty of Medical & Health Sciences, University of Auckland, Auckland, New Zealand

**Keywords:** Anorexia nervosa, Adolescent, Treatment

## Abstract

Recovery and remission rates of adolescent anorexia nervosa (AN) following Family Based Treatment (FBT) have seen a relative decline over recent years. While reasonably successful in achieving physical recovery (i.e. weight restoration), both empirical and anecdotal accounts highlight a lack of attention to the psychological recovery of the adolescent within manualised FBT. As such, there is a need for innovation to explore treatment variations and alternatives for the proportion of adolescents with AN who do not respond favourably to this first-line treatment. This paper introduces a new treatment framework to the field for clinical consideration and empirical assessment. Adolescent and Parent Treatment (APT) for adolescent AN draws from both family-based and individual treatment models, applying a developmental lens. APT attends to physical and psychological recovery simultaneously and from the start of treatment, with capacity to tailor individual psychological modules to the adolescent formulation. While clearly in its infancy, APT provides an exciting new avenue for exploration within the field, as we seek new avenues to support young people and their families to effectively combat this deadly illness.

## Background

Anorexia Nervosa (AN) is a devastating illness with limited treatment response [1] and significant biopsychosocial consequences for the developing adolescent [2, 3]. For the past few decades, a specialist form of family therapy (FT) has been the treatment of choice for adolescents with AN. A formative randomised controlled trial from the Maudsley Hospital in London highlighted superior response for adolescents (< 18 years) receiving FT-AN compared to an individual supportive therapy, where illness duration was under 3 years [4]. This finding held at 5 year follow-up, where approximately 2/3 of adolescents were considered recovered [5]. NICE guidelines recommended FT as first-line treatment for adolescent AN, resulting in widespread dissemination of the approach both in its original (FT-AN) and manualised forms (Family-based Treatment or FBT) [6].

Manualised FBT is to date the most widely studied treatment for adolescent AN, and has equipped countless families to support their adolescent toward recovery. Across the six randomised controlled trials (RCTs) of FBT in adolescent AN to date, partial response to treatment (improvements in weight and AN symptoms) varies from 60 to 85%, with remission from AN (weight > 94% body mass index (BMI) & Eating Disorder Examination-Questionnaire (EDE-Q) within 1 standard deviation of population means) found in 22–49% of cases [7]. Viewed chronologically, these studies evidence a drop over time in the efficacy of FBT when compared to the original Maudsley FT-AN study (where 2/3 recovered). This outcome data is complicated by varied use of recovery and remission criteria across sites and trials, as noted by an Australian study where between 21.7–87.7% remission for their cohort was found, depending on which criteria was applied [8]. It should be noted that a drop in treatment efficacy over time is not new in psychotherapy research, as evidenced by the CBT for depression literature [9]. Nonetheless, there is consensus within the field that even with strict adherence to this manualised treatment model, FBT can only be considered effective in achieving full recovery for just under half of treated adolescents [7].

From a service-user perspective, a small number of studies report mixed experiences of treatment in the context of FBT. Acceptability of outpatient FBT post-treatment has been reported as highly positive by 82% of parents, and 59% of adolescents [10]. High parental ratings have also been found at 5 year follow-up of an inpatient cohort [11], where again retrospective ratings from adolescents were significantly lower. Strong retrospective ratings of FBT by families have also been found regarding assessment, education, clinical team interaction, and achievement of physical health [12]. Alongside this, the literature is beginning to highlight some negative impacts of treatment where FBT is a poor fit to the family. For example, adolescents feeling unsupported/unheard and negatively toward their FBT therapist [12], treatment reinforcing parents’ own sense of guilt and blame [13], and dilemmas experienced by therapists where FBT adherence is valued by supervisors over clinically-informed adaptation [14]. Former patients of a family-based inpatient treatment setting describe (for example) a sense of being treated as a number (on the scales) not a person, and experiencing clinicians rigid adherence to treatment protocols rather than consideration of the patients as individuals [15]. The theme of a perceived need to shift treatment focus from weight alone to a more holistic, individualised balance between physical and psychological recovery is replicated in the patient and parent voice in different countries [16].

The approach to adolescent AN treatment in the past few decades has held a number of assumptions. Of particular note, it has been assumed that a malnourished state compromises cognition to the point where individual work is contra-indicated, and that the strength of the “AN voice” means parents must hold full agency around food & eating [6]. While often apt, clinician experience and more detailed empirical assessment highlights that adolescents do not universally present with poor insight and low motivation to change [17], yet treatment approach has not allowed flexibility in this regard. Additionally, it is worth considering whether prioritising adherence over non-intervention factors such as alliance, therapist experience [18], and fostering a more collaborative family system, is serving treatment outcome & experience. As a field, we must remember that evidence-based practice comprises not simply of delivering treatment models supported by research, but “the integration of the best available research with clinical expertise in the context of patient characteristics, culture, and preferences” [19]. Balancing the so-called “three-legged stool” across research evidence, clinician factors, and patient factors is essential if we are to move closer to effective outcomes [20].

Both the aforementioned remission rates, anecdotal clinical impression, and service user perspectives highlight that refeeding alone is not sufficient for an adolescent to fully recover from AN. While FBT researchers turn their attention to critical questions around moderators and mediators of treatment response in order to inform effective triage of FBT-appropriate families [21, 22], as clinicians we are left wondering what else there is to offer the families for whom FBT is ineffective, unfeasible, or unacceptable. To this end, a number of FBT variants, augmentations & adaptations (such as multi-family therapy, parent-focussed treatment) have been explored across multiple treatment settings (for a review, see 23), with emerging efficacy evidence to date generally in line with standard FBT. Much work is underway in this space. Only recently, individual psychological intervention for adolescent AN is again gaining interest.

A number of cohort studies/case series have utilized a specialised form of individual cognitive behavioural therapy (CBT) for adolescent AN, including enhanced-CBT (CBT-E) from the adult literature [23], with minor modifications for adolescents. Adolescent CBT/−E is showing both good completion rates and positive outcomes, with an Italian study reporting that of those completing CBT-E (63%), an average of 8.6 kg weight gain was achieved, and almost all (96.6%) showed only minimal residual symptoms post-treatment as measured by the Eating Disorders Examination-Questionnaire (EDE-Q < 1 SD above community norm [24];. Replication in a community setting similarly found 62.9% of treatment completers (completers being 71.4% of the 49 enrolled) showed “full response” (both weight recovery to > 18.5 BMI, and EDE-Q < 1SD above the community norm) [25]. When considering the full intent-to-treat cohort, those with a “good BMI outcome” (> 18.5 BMI) drops to 45% at end of treatment, placing weight recovery outcomes in line with FBT. It should be noted that these case series are not comparative trials and do not have the scientific rigour of the RCT design as in the FBT trials discussed above. In a step toward directly comparing these treatments, a recent non-randomised effectiveness trial compared CBT-E with FBT in adolescent AN [26]. Results showed no difference in EDE-Q scores over time across treatment groups, but faster initial weight recovery for lower weight adolescents whose families opted for FBT. This advantage of FBT no longer held at 6- or 12-month follow-up. Interestingly, baseline EDE-Q global score was notably lower (1.9/6) in the FBT compared to the CBT-E group (3.3/6; Cohen’s *d* = 1.04) suggesting that the FBT group presented with a less severe illness which likely also influenced faster weight recovery. These findings lend support to elements of both treatments, in particular the benefit of parental input for strong initial weight recovery, yet also the effectiveness of individual work (adolescent agency) in still achieving & maintaining weight restoration.

In considering the content of the different treatment approaches, adolescent CBT-E requires agreement to recovery from the adolescent, and adolescents are given a choice regarding parental refeeding. This excludes an important recovery component offered by FBT (i.e. parent involvement to actively facilitate prompt refeeding). Therefore, whilst adolescent CBE-E shows early promising results that a more psychologically focussed individual treatment may be effective, it is likely that outcome data to date largely excludes those adolescents who are precontemplative about treatment and recovery, limiting the generalisability of results. Moreover, a limitation of adolescent CBT-E is that the individual therapeutic content is essentially the same as that designed for adults, lacking the benefit of a developmental lens.

Taken together – parental refeeding, and individual psychological work - it is suggested that concurrently focussing on psychological recovery (through individual treatment) alongside essential physical recovery (parent lead refeeding of the adolescent) may hold the potential for improved treatment engagement and recovery outcomes for this vulnerable young group. This paper seeks to highlight a new idea to the field, for clinical consideration and empirical assessment.

## The new idea

Adolescent and Parent Treatment (APT) is a recently developed treatment option drawing from both family and individual treatment approaches for adolescent AN, together with empirical understanding of predisposing and maintaining factors of AN. It addresses both physical and psychological recovery simultaneously and from the start of treatment through a) intensive parental refeeding with the support of the therapist and a dietician, and b) four targeted individual treatment modules that address psychological, emotional, temperamental, and developmental needs of the adolescent.

### The genesis of APT

APT has its roots in Ego Oriented Individual Treatment (EOIT), an individual treatment developed during the 1980/90’s in Michigan, USA. In EOIT the adolescent receives individual psychodymanic psychotherapy focused on ego strength, coping style, individuation and identity development. Importantly, parents are provided with education and input from the dietitian to support refeeding efforts, and the therapist provides parents with psychoeducation and strategies to understand how to support their child. In a seminal controlled comparison with a family systems behavioural therapy in a cohort of 37 adolescents with AN, both EOIT and the family systems treatment performed comparably on physical and psychological outcomes at both end of treatment and at 12-month follow-up [27], with the family systems group producing faster weight recovery.

A decade later, EOIT was modified to act as a comparison treatment for manualised FBT in a larger-scale RCT [28]. The modifications excluded parental refeeding and dietetic support, thereby creating a pure individual therapy (matched in dose to FBT) that was re-labelled Adolescent-focussed Treatment (AFT). FBT outperformed AFT in weight recovery at end of treatment (*p* = 0.048). While no significant difference between groups achieving “full remission” (95% IBW & EDE-Q score within 1 SD) at end of treatment was found (42% FBT; 23% AFT), more in the FBT group sustained full remission (but not partial remission) at both 6- (40%/18%) and 12-month follow-ups (49%/23%). Interestingly, at 2-year follow-up the differences in remission rates between the groups had become negligible, as the AFT group continued to improve (9 new AFT adolescents achieved full remission; 1 FBT adolescent) whilst relapse was observed in the FBT group [29]. These findings highlight the potential role of individual treatment to address underlying maintaining/ predisposing factors of the AN and in turn, a likely positive impact on relapse prevention.

Given the need to search for alternative treatment options, in recent years the original EOIT treatment framework has been revisited and substantially modified by the authors (MG, LA) in collaboration with the original EOIT developer (Dr Ann Moye). The role of parents in re-feeding has been strengthened and updated in line with learnings from the FBT/FT-AN literature, and through the re-introduction and refinement of dietetic interventions. Similarly, the individual adolescent modules have been substantially enhanced, drawing from the fields’ updated understanding of the role of biological/temperamental factors, together with other typical predisposing and maintaining factors, all set in the context of the tasks of adolescent development. Given the integrative model focusing on both the parent role and adolescent therapy, the treatment has been termed Adolescent and Parent Treatment (APT).

### The framework of APT

APT is a manualised (unpublished) yet highly flexible treatment, consisting of approximately 20–30 treatment sessions over 9–12 months. APT utilises a collaborative and patient centred model of care, whilst simultaneously taking into account the developmental age/needs of the adolescent, the severity of the anorexia, the level of insight/motivation, and the capacity of the parent/s. It is an appropriate framework to use across both adolescence and emerging adulthood (~ 12–24 years old) given its adaptability both in terms of parent role and content delivery to the developmental phase. APT is conceptualised into three active treatment phases. Key content covered with parents and adolescents is summarised in Tables 1 & 2.

**Table 1:** Content of APT parent treatment modules

1. Psychoeducation	Provides psychoeducation regarding AN and impact on their adolescent
2. Re-feeding	Focuses on setting up and maintaining renourishment requirements
3. The AN cycle	Helps parents understand their adolescent’s psychological functioning and how this contributes to and maintains the AN
4. Managing distress	Covers all aspects of managing their adolescent’s distress and AN behaviours
5. The parent role	Helps parents understand and reflect on their own attributes and strengths and weaknesses that impact treatment

**Table 2:** Content of APT adolescent treatment modules

1. Standing up to AN	Understand the physical and psychological impact of AN on their brain and body Learn to externalise AN, let go of AN, and replace AN with more appropriate methods Learn how to manage AN voice and cognitions, and how to “stand up” to AN
2. Managing Emotions	Identify and understand their emotions Learn strategies to manage and self-regulate their emotional states
3. Building a core sense of self	Learn to understand how they function and importance of developing a core sense of self Exploring their personality traits, inner critics, and how to build and utilise their inner resources (sages)
4. Developmental Challenges	Exploring adolescent developmental tasks and understand how AN impacts or de-rails these tasks Psychoeducation on physical and neurological development Psychoeducation on developmental needs such as managing social connectedness and relationships Discovering how to promote neural plasticity through curiosity and exploration

#### Phase 1 of APT

Phase 1 consists of assessment, establishing parental refeeding (parent sessions), and fostering adolescent rapport and engagement (adolescent sessions). This phase consists of approximately 6–8 weekly sessions post-assessment. Where feasible sessions in phase 1 are up to 90-min long, allowing 30 min for adolescent work, 30 min for parent work, and 30 min to bring the family together to clarify the coming weeks goals. Alternatively parent and adolescent sessions can be delivered separately, with the parent joining at the end of the adolescent session.

Assessment entails gaining a thorough understanding of both the adolescent (e.g. AN symptomology, comorbidities, traits, psychosocial/developmental functioning, attachment style, motivation to change) and the parents/family system (parenting style, parental cohesion, family food culture, attunement toward child). This leads to an individualised case formulation, which is communicated to the family and guides treatment.

Parental refeeding is established promptly and, as with FBT, is a key focus of phase 1 (see Table 1). APT does not assume that all parents have the same capacities and knowledge to refeed their child. Setting the refeeding expectations too high can become distressing for many parents resulting in sense of failure, avoidance, empathic distress, and/or diminished self-efficacy [13, 16]. APT also acknowledges the negative impact for adolescents who are continually exposed to parental refeeding failures or inconsistencies, and the resulting high expressed emotion [30].

Rather, parental responsibility of refeeding is collaboratively discussed and agreed to at the commencement of APT, with the level of parental responsibility matched to each family. This is based on both the parental capacity to undertake refeeding, and the adolescent’s level of insight and commitment to recovery. In general, parental responsibility is strong where possible (particularly with younger adolescents) at the start of treatment, with parents educated & equipped to effectively refeed the adolescent in parent sessions with the therapist. Four parent dietetic sessions are available to support nutritional education and parental confidence to refeed adequately. Parents are also provided with an APT skills training manual to guide them through treatment alongside parent sessions.

The adolescent is made aware of the need to modulate responsibility for refeeding throughout treatment, in that steady weight recovery allows responsibilities to be gained by the adolescent, but weight loss requires responsibilities returning to the parents. Such modifications are reviewed during session on a regular basis, with timeframes articulated by the therapist & tailored to each family. APT therefore individualises refeeding to both adolescent and parent in an effort to reduce conflict and maintain a strong parent/child alliance.

Parents are further supported through general eating disorder psychoeducation, externalizing the AN, and teaching parent/child co-regulation strategies. Such strategies are designed to foster parent/adolescent attunement and parental management of their adolescent’s distress, therefore supporting refeeding efforts.

The second key focus of phase 1 is individual work with the adolescent (or with parent staying for younger adolescents in line with their preference) to build rapport & provide general eating disorder psychoeducation and support. This involves psychoeducation, establishing a therapeutic alliance by demonstrating a genuine interest in young person beyond their eating disorder, and motivating the young person to be an active participant in their treatment (drawing from motivational interviewing [31]). These factors have been reported by adolescents to be the most helpful aspects of treatment [32] and provide a stable therapeutic relationship from which to begin the psychological work of phase 2. Adolescents are ready for phase 2 when weight recovery is well established, and both rapport and motivation for recovery are evident.

#### Phase 2 of APT

**Fig. 1:**
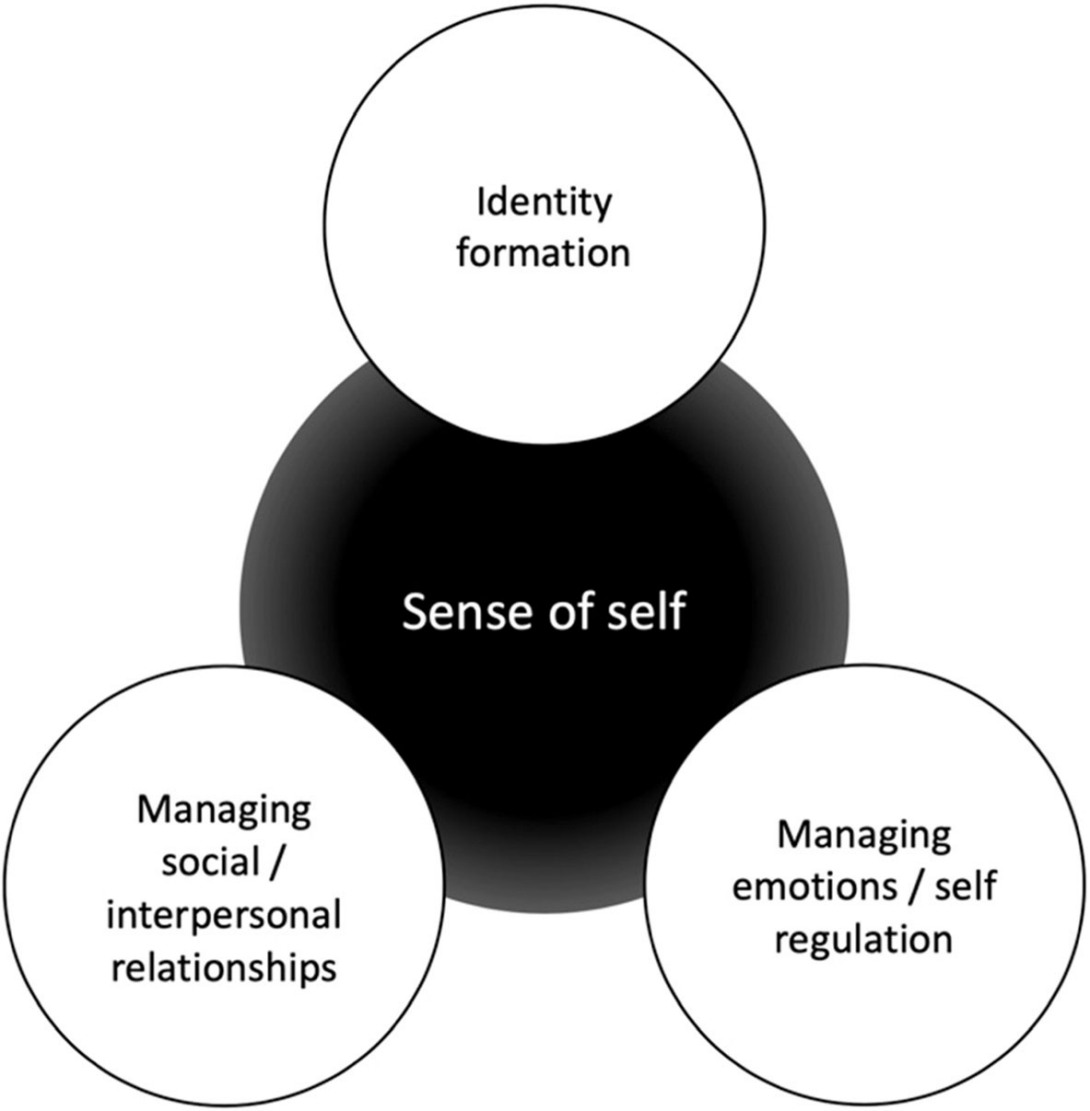
Core components of the adolescent psychological treatment modules in APT

Phase 2 encompasses the core components of the adolescent psychological treatment (see Table 2 and Fig. 1), alongside ongoing refeeding. Sessions are still weekly but now 60-min long, with more time allocated to adolescent work. Sessions can be co-joint or separate depending on the family’s needs and the formulation, but typically parent involvement in treatment will phase out as phase 2 progresses (e.g. joining for last 10 min of the session). Modulation of refeeding responsibility is ongoing, with the goal of the adolescent picking up autonomy with ongoing progress in recovery.

Design of the individual adolescent component takes into account the adolescent’s age and developmental trajectory, personality traits, coping style and co-morbidities. Adolescents 16 years plus and emerging adults (~ 18–24 years) are generally seen without their parents for the adolescent work, given their differing developmental needs of individuation and identity formation [33]. Clinician stance is supportive, collaborative and compassionate.

As outlined in Table 2, there are 4 key psychological components that involve psychoeducation, self-reflection through journaling and therapeutic discussion, and new skill development. The 4 key tasks are 1) Standing up to AN (externalisation, identifying and re-framing the AN voice, replacing AN with more adaptive coping strategies), 2) Managing emotions (understand emotional states, drawing from the polyvagal theory [34] to shift from state of threat (AN) to a state of safety & connection, leading to greater self-regulation), 3) Building a core sense of self (understanding, exploring and accepting own personality traits, inner critics, and inner resources/values), and 4) Developmental challenges (navigating physical/neurological development including body confidence, managing relationships, exploration of interests). These components are prioritised based on individual formulation, and conceptualised as AN disrupting 3 key tasks of adolescence: identity development, managing emotions, and building relationships (see Fig. 1). It is of note that there are similarities in focus of the first 3 psychological components of APT and those articulated in the Maudsley Maintenance Model of Adult Anorexia Nervosa [35] and its resulting treatment protocols (MANTRA [36] & FREED [37]). While content topics overlap, the treatment approaches are distinct in delivery due to APT consistent application of developmental stage to the pitch and delivery of the therapeutic content.

To support and guide both the adolescent and clinician throughout phase 2 but particularly in developing a core sense of self, three key resources are utilised: Unpack Your Eating Disorder Workbook [38], Letting Go of ED - Embracing Me Journal [39], and the Trait, Critic and Sage Therapy Cards [40]. The transition to phase 3 occurs when the APT psychological content appropriate to the adolescent has been covered, weight is largely restored, and the adolescent is functioning on an age-appropriate developmental trajectory.

#### Phase 3 of APT

In phase 3, the focus is on consolidating gains made in treatment, providing ongoing monitoring/accountability, and addressing any additional underlying maintaining factors based on adolescent formulation such as clinical perfectionism, developing assertive communication skills, or anxiety work. There should be minimal AN cognitions or behaviours, and the adolescent is expected to manage their own nourishment and any comorbidities. Sessions are fortnightly to monthly, with flexibility in the number of sessions required depending on formulation (e.g. 5–10). A relapse prevention plan is developed prior to discharge.

## State of evidence & future directions

APT draws from broader empirical knowledge and treatment evidence for adolescent AN, but as yet remains untested as a package of treatment in its current form. Support for the feasibility and early efficacy of APT is anecdotal only to date, largely based on the clinical experience of the authors and their colleagues. Given that APT is a new idea, next steps in the assessment of APT as a treatment package should first focus on presenting case reports to the literature, followed by high quality non-comparative observational studies (e.g. case series) in real world clinical settings where APT is being delivered. In line with recent recommendations, careful attention should be paid to thorough assessment of clinical features at baseline and end of intervention to assess change, with follow up of adolescent outcome tracked as long as possible [41]. For these observational studies to be robust, both initial training and ongoing competency development/supervision of clinicians will be required in order to ensure adherence to the APT framework. Alongside case series, well designed investigations of feasibility [42] will allow us to understand the strengths and weaknesses of the intervention idea, for example in terms of acceptability (patients, carers, clinicians), demand, implementation, and practicality, alongside limited-efficacy testing of treatment response. Once this initial work has been done, and assuming outcomes are promising, APT can then be considered for a non-randomised effectiveness trial before proceeding to direct comparison with other adolescent AN treatments in a RCT design.

## Conclusions

APT is proposed as a novel treatment variant for adolescent AN drawing from the strengths of both family-based and individual treatment approaches, that merits early empirical investigation as a standalone treatment package in the outpatient setting. The combined approach of both active physical recovery (family work) and active psychological recovery (individual adolescent work within a developmental frame) simultaneously and from the start of treatment is uncommon in the adolescent AN “evidence-based” treatment space, where typically an either-or (family or individual) approach is taken. While clearly in its infancy, APT provides an exciting new avenue for exploration within the field, as we seek new avenues to support young people and their families to effectively combat this deadly illness.

## Data Availability

Not applicable.

## Abbreviations

AN: Anorexia nervosa
FBT: Family-based treatment; FT Family therapy; FT-AN Family therapy for anorexia nervosa
APT: Adolescent-parent treatment
BMI: Body mass index
CBT: Cognitive behavioural therapy
EDE-Q: Eating Disorders Examination-Questionnaire
EOIT: Ego Oriented Individual Treatment
RCT: Randomised Controlled Trial
